# Exploring the Impact of Dentine Hypersensitivity: Validation of the Spanish DHEQ-15 and Its Cultural Adaptation

**DOI:** 10.4317/jced.62803

**Published:** 2025-09-01

**Authors:** Tomiris Bazarova, José María Montiel-Company, Francisco José Gil-Loscos

**Affiliations:** 1 University of Valencia, Stomatology Department, Valencia, Spain. Orcid-ID. 0009-0006-4199-834X; 2 University of Valencia, Stomatology Department, Valencia, Spain

## Abstract

**Background:**

Dentine hypersensitivity (DH) is a common condition with varying patient perceptions, making diagnosis and management challenging. Patient-reported outcome measures, such as the Dentine Hypersensitivity Experience Questionnaire (DHEQ-15), provide valuable insights into its impact on daily life. This study aimed to adapt and validate the Spanish version of the DHEQ-15 for use in Spain, ensuring its reliability and relevance for Spanish-speaking patients.

**Material and Methods:**

A structured translation and cross-cultural adaptation process followed Beaton *et al*.’s (2000) methodology. Spanish-speaking participants aged 35–60 completed the DHEQ-15. Psychometric properties were assessed through factor analysis for construct validity, Cronbach’s alpha for internal consistency, and the intraclass correlation coefficient (ICC) for test-retest reliability. Sociodemographic factors such as gender, age, and education level were also analyzed.

**Results:**

The Spanish DHEQ-15 was well-received. It showed excellent internal consistency (Cronbach’s alpha = 0.958) and high test-retest reliability (ICC = 0.932). Factor analysis identified three main dimensions: Restrictions-Coping, Psychosocial Impact, and Identity explaining 76% of score variance. The questionnaire was easy to complete, with an average completion time of 4 minutes. Women scored higher in ‘Restrictions-Coping,’ while age and education level showed no significant associations.

**Conclusions:**

The Spanish DHEQ-15 has been successfully adapted and validated in Spain, demonstrating high reliability and validity. It is a viable and effective tool for assessing the impact of DH on the quality of life of Spanish-speaking populations.

** Key words:**Dentine hypersensitivity, Validation, Quality of life, Questionnaire.

## Introduction

Dentine hypersensitivity (DH) is characterized by short and sharp pain arising from exposed dentine in response to stimuli, typically thermal, evaporative, tactile, osmotic, or chemical, which cannot be ascribed to any other dental defect or pathology [1].

DH is a common oral complaint, with a global prevalence estimated at 33.5%, according to a 2019 systematic review and meta-analysis that included 77 studies, showing considerable variability in the results [2]. This variability in prevalence is not only due to different diagnostic methods used but also to various factors that directly impact the development of the condition. These factors include age, gender, and underlying conditions such as periodontal disease. Younger populations, particularly those aged 18 to 35, exhibit higher prevalence rates due to dietary habits and parafunctional behaviors that lead to dental wear and dentine exposure [3]. Additionally, DH tends to decrease with age, as secondary dentine formation and the sclerosis of dentinal tubules provide increased protection. Although studies on gender differences are inconclusive, some suggest that women may experience a higher prevalence of the condition, potentially due to more rigorous oral hygiene habits, even though these differences don’t always reach statistical significance [4]. Furthermore, after periodontal treatment, the prevalence of DH increases significantly, affecting 55% of patients in the first few weeks, which notably impacts their quality of life [5].

Pain is the main symptom of the condition, significantly disrupting daily activities such as talking, eating, drinking, toothbrushing, and social interaction [6]. However, many individuals do not consider DH a serious oral health issue and, therefore, do not seek professional treatment. In some cases, the condition is only mentioned during dental visits as a secondary concern rather than the main reason for the appointment. The severity of pain differs from person to person, with some experiencing only mild discomfort while others find it more disruptive [7]. As the importance of a patient-centered approach in healthcare continues to grow, clinicians and researchers have come to recognize that a patient’s perspective is just as important as clinical outcomes when diagnosing or evaluating the success of a treatment. This shift has driven the growing emphasis on patient-reported outcomes, particularly the concept of health-related quality of life (HRQoL). Within this framework, oral health-related quality of life (OHRQoL) is crucial for understanding how oral health affects an individual’s overall well-being, including their ability to function, psychological state, social interactions, and the pain or discomfort they experience [8].

In recent years, numerous tools have been developed to evaluate OHRQoL, including general questionnaires like the GOHAI, OHIP, and OIDP. However, these broad measures often fail to capture the specific impact of individual oral conditions on OHRQoL. In contrast, condition-specific instruments offer a more precise understanding of the unique effects of a particular condition, allowing for a clearer assessment of its influence on a patient’s well-being. To address this gap, Boiko *et al*. (2010) developed the Dentine Hypersensitivity Experience Questionnaire (DHEQ), focusing specifically on evaluating the impact of DH on OHRQoL [6].

The DHEQ consists of 48 items; however, only 34 contribute to the impact scale used to assess the effects of dentine hypersensitivity on OHRQoL. This impact scale includes five domains: Restrictions, Coping, Social Impact, Emotional Impact, and Identity [6].

Responses are given on a seven-point Likert scale, ranging from ‘strongly disagree’ to ‘strongly agree.’ The measure has demonstrated excellent reliability, validity, and responsiveness over time, and can effectively discriminate between treatments of varying efficacy [6,9]. While the DHEQ has proven to be a reliable and valid tool for assessing the experience of DH, it can take a long time to administer and complete, being ineffective for clinicians.

Machuca *et al*. (2013) developed and validated a shorter version of the DHEQ, called the DHEQ-15, which consists of 15 items. The DHEQ-15 retains the same five subscales as the original DHEQ, along with the 7-point Likert scale for responses. The total impact score is calculated by summing the responses across all 15 items. Higher scores indicate a negative impact of dentine hypersensitivity on an individual’s quality of life. The DHEQ-15 also demonstrates strong psychometric properties and is recommended for identifying individuals with dentine hypersensitivity, as well as evaluating treatments [10].

Since its initial development, the DHEQ-15 has been translated and adapted into Chinese [11], Brazilian Portuguese [12], Romanian [13], Arabic [14], and Turkish [15]. Cross-cultural adaptation of an instrument is crucial when introducing it to a new country, culture, or language to ensure semantic, idiomatic, experiential, and conceptual equivalence between the original and target versions [16]. Therefore, this study aimed to adapt and validate the DHEQ-15 for use in the Spanish language.

## Material and Methods

-Sample Size and Selection

The sample size was determined based on recommendations for psychometric validation studies, which suggest recruiting at least ten participants per questionnaire item [17]. Participants were included if they were native Spanish speakers and between 35 and 60 years old, regardless of whether they experienced dentine hypersensitivity. Exclusion criteria included prior participation in DH-related studies, the presence of advanced periodontal disease (mobility greater than grade I), or sensitive teeth due to erosion, abrasion, or recession of exposed dentine.

- Data Collection

Before participation, individuals received an information sheet explaining that the study was voluntary and anonymous. The questionnaire was administered digitally via the online platform LimeSurvey and distributed to the target population between July and October 2022. The questionnaire link was primarily shared via WhatsApp with patients from the University of Valencia dental clinic and other private practices.

- Development of the Spanish version of DHEQ-15

The DHEQ-15 was cross-culturally adapted and validated into Spanish using the forward-backward translation process proposed by Beaton *et al*. [16]. This process includes the following five stages:

1. The DHEQ-15 was translated from English to Spanish by two independent translators whose native language is Spanish with expertise in healthcare. One of the translators was familiar with the objectives and concepts of the questionnaire.

2. These two versions were synthesized into a single version after a discussion between the translators.

3. The synthesized version was back-translated from Spanish to English by two professional translators who are fluent in Spanish but whose native language is English. Neither translator was familiar with the original instrument.

4. The translated, synthesized, and back-translated versions were compared and discussed by an expert panel consisting of translators and members of the research team.

5. The preliminary Spanish version of the DHEQ-15 was pilot-tested on a convenience sample of 15 individuals who were not included in the subsequent psychometric validation. Each participant was interviewed to identify any difficulties in completing the questionnaire or understanding the meaning of its items and responses. Based on the results of the pilot study, the final version of the DHEQ-15 Spanish version was created by the expert panel ( Table 1).

The DHEQ-15-Esp consists of 15 items measured on a 7-point Likert scale, ranging from 1 (‘strongly disagree’) to 7 (‘strongly agree’). The total score is obtained by summing all item responses, resulting in a range from 15 to 105, with higher scores indicating a greater negative impact of DH on quality of life.

- Statistical Analysis

The data were statistically analyzed using SPSS 24.0. Differences in DHEQ-15 scores across demographic characteristics were assessed using an independent t-test for comparisons between two groups and one-way ANOVA for comparisons among multiple groups. Statistical significance was set at *p* < 0.05.

- Reliability analysis

When evaluating the reliability of the scale, two aspects should be considered: internal consistency and test-retest reliability. Internal consistency was assessed using Cronbach’s alpha coefficient to determine how well the questionnaire items measure the same concept [18]. The values of alpha ranging from 0.70 to 0.95 were considered acceptable [19]. Each item’s contribution to the overall scale was analyzed by calculating the corrected item-total correlation. Additionally, the impact of removing specific items on Cronbach’s alpha was examined to maintain the coherence of the questionnaire.

Test–retest reliability was determined by calculating intraclass correlation coefficient (ICC). Participants completed the questionnaire twice, with a three-week interval between administrations, allowing for the assessment of response stability. The ICC was used to measure consistency, with values greater than 0.90 indicating excellent reliability [20].

- Validity analysis

The Exploratory Factor Analysis (EFA) was used to identify domains of the DHEQ-15. Principal component extraction was performed using Varimax rotation with Kaiser normalization. The adequacy of the dataset for factor analysis was confirmed through Bartlett’s test of sphericity and the Kaiser-Meyer-Olkin (KMO) measure. To further refine this analysis, Confirmatory Factor Analysis (CFA) was employed to validate the factor structure identified in the EFA.

## Results

The expert panel reviewed the translations and confirmed that the items accurately reflected the original meaning, maintained the intended idiomatic expressions, and did not pose translation challenges. The piloting of the pre-final version of Spanish DHEQ-15 indicated that the questionnaire was easy to complete in an average of 4 min, and all items were relevant and clearly understood. However, the panel identified difficulties with items 11 and 12, as they did not reflect the same meaning as the original English version. Consequently, the translations were modified: item 11 was associated with mood, while item 12 was linked to a localized sensation in the mouth.

Patients’ characteristics

The study included a total of 205 participants. The distribution of sociodemographic characteristics is shown in  Table 1. The mean age of the sample was 51.4 ± 6.5 years (range 35–60), with the majority of participants being female (55.6%). In terms of education level, the largest proportion had higher education (65.8%), while the smallest percentage had secondary education (3.4%), and no participants were classified as illiterate, ( Table 2).

- Reliability

The overall Cronbach’s alpha for all 15 items was 0.958, indicating high reliability. The overall questionnaire statistics, including the alpha coefficient if each item is deleted, ranged from 0.953 to 0.959. This suggests that removing any item would not improve the internal reliability of the questionnaire.

Test-retest reliability of the DHEQ-15 Spanish version was evaluated with 15 participants over a 3-week interval. The Pearson correlation coefficient was 0.933, and the ICC was 0.932, both indicating excellent reproducibility and consistent measurements, ( Table 3).

- Validity

The KMO test value was 0.949, and Bartlett’s test of sphericity was 2708.354 (df = 105, *p* ≤ 0.001), indicating the suitability of the data for Exploratory Factor Analysis (EFA).

In the EFA, with a minimum eigenvalue set at 0.5, we found that five factors explained 83.5% of the total variance, aligning with the five dimensions of the DHEQ-15-Sp. When considering the questionnaire as a whole, without breaking it down into dimensions, it explained 63.4% of the variability.

The factor loadings after Varimax rotation revealed three main domains: the first domain includes items related to Coping, Social, and Emotions; the second combines Identity and Emotions; and the third groups Restrictions and Coping. However, items 3, 9, and 12 formed separate, less-defined domains.

To further refine the construct validity, a Confirmatory Factor Analysis (CFA) was conducted with a forced three-dimensional structure ( Table 4). The results suggest that three domains best represent the DHEQ-15-Sp. The first domain, Restrictions and Coping, consists of items 1–8, incorporating items 7 and 8 from Social impact, which are better aligned as adaptations in daily habits to manage DH. The second domain, Identity, includes items 13–15, preserving its original structure. The third domain, Social and Emotions, was renamed Psychosocial Impact and encompasses items 9–12.

Once the three domains were defined, the internal consistency of each was assessed. Cronbach’s alpha values ranged from 0.882 to 0.943 for the three-factor structure, with the following results for each domain: Restrictions-Coping (Cronbach’s alpha = 0.943), Psychosocial Impact (Cronbach’s alpha = 0.880) and Identity (Cronbach’s alpha = 0.882).

- Relationship Between DHEQ-15-Esp and the Variables of Gender, Age, and Professional Level

The results show a significant difference in the Restrictions-Coping domain, where women (mean 29.45) reported greater difficulties than men (mean 26.17), with a *p-value* of 0.019. However, no significant differences were found in the Psychosocial Impact or Identity domains, as well as in the total score.

On the other hand, no significant differences were observed regarding age and educational level, with *p-value*s greater than 0.05, suggesting that the impact of DH is similar across these groups.

## Discussion

This study aimed to perform the cross-cultural adaptation and validation of the Spanish version of the Dentine Hypersensitivity Experience Questionnaire (DHEQ-15). The decision to adapt a questionnaire to the Spanish culture is based on the fact that developing new instruments is a complex and time-consuming process. In contrast, cross-cultural adaptation is more efficient and ensures measurement equivalence [21]. Therefore, this is the first study to adapt and validate the DHEQ-15 in Spain, providing a reliable tool to measure the impact of DH in the Spanish population.

The Spanish DHEQ-15 demonstrated a high level of reliability, with an internal consistency coefficient of 0.953. This value is higher than the Arabic (0.912) [14], English (0.906) [10], Chinese (0.934) (11), and Brazilian (0.945) [12] scales. The Turkish DHEQ-15, however, exhibited an even higher reliability with a coefficient of 0.970 [15]. Considering that a value between 0.70 and 0.80 is generally considered adequate according to Bland and Altman’s recommendations [19], the internal consistency coefficients in all of these versions are above the minimum value of 0.9, demonstrating strong reliability across different cultural and linguistic contexts.

The test-retest reliability value for the Spanish version was found to be 0.932, indicating an excellent correlation, consistent with the values reported for the Chinese (0.894) [11], Arabic (0.914) [14], and Turkish (0.920) [15] scales. These results suggest that the Spanish DHEQ-15 can be considered a reliable and stable tool for assessing dentine hypersensitivity.

Cultural validity was demonstrated in this study, with the Spanish DHEQ-15 exhibiting a three-factor structure, consistent with the three-factor structures of the Chinese and Arabic adaptations, and differing from the single-factor structure of the Turkish version and the five-factor model of the Brazilian and original DHEQ-15 versions.

The original version includes five domains: ‘Restrictions’, ‘Coping’, ‘Social’, ‘Emotions’, and ‘Identity’ (6,10). In contrast, the Chinese and Arabic versions restructure these domains into three. Specifically, the Chinese version merges ‘Coping’ and ‘Social’ into ‘Changes in Eating Habits’ and combines ‘Emotions’ with ‘Identity.’ The three domains in the Chinese version are ‘Restrictions’ (items 1–3), ‘Changes in Eating Habits’ (items 4–9), and ‘Emotions and Identity’ (items 10–15).

The Arabic version follows a similar structure, except for item 10 (anxious about eating contributes), which is categorized under ‘Changes in Eating Habits’ instead of ‘Emotions and Identity’ due to its focus on dietary concerns. Additionally, items 5 (“careful when breathing”) and 9 (“painful at the dentist”) were considered unrelated to eating habits, leading to the suggestion that this domain be renamed ‘Changes in Habits.’

Our Spanish version also follows a three-factor structure but with a different distribution: ‘Restrictions and Adaptations’ (items 1–8), merging the original ‘Restrictions’ and ‘Adaptations’ domains while incorporating items 7 (“taking longer than others to finish”) and 8 (“choosing food with others”) from ‘Social.’ This reclassification is more logical, as these questions can be considered adaptations in daily habits to manage DH. The second domain, which we renamed as ‘Psychosocial Impact,’ includes items 9–12 and reflects the psychological and interpersonal effects of DH, while the third domain, ‘Identity’ (items 13–15), retains its original structure. These variations underscore the influence of cultural and linguistic factors on how individuals perceive and categorize their experiences with DH, ultimately shaping the questionnaire’s factor structure.

The relationship between the DHEQ-15-Sp and variables such as gender, age, and education level did not show any statistically significant differences. In terms of gender, the results indicate that women experience greater difficulties in the ‘Restrictions-Adaptations’ and ‘Psychosocial Impact’ domains, suggesting a higher degree of dentine hypersensitivity and a potential association with being female. No significant variations were found with age or education level, suggesting that the perception of dentine hypersensitivity’s impact is not influenced by these factors. In the Turkish DHEQ-15 study, it was found that women and individuals with lower levels of education are more susceptible to DH. These demographic insights could inform clinical practice by encouraging healthcare providers to take gender and education into account when developing treatment strategies for DH [15].

The present study has several limitations that should be acknowledged. First, selection bias may be present due to the online nature of the survey, which could limit the representativeness of the sample by excluding individuals without access to or familiarity with digital technology. Additionally, the use of self-reported responses may affect the accuracy of the data. Another potential source of bias arises from the distribution method, as the questionnaire link was primarily shared via WhatsApp. Lastly, the validity of the questionnaire was not assessed in individuals under 35 or over 60 years of age, which may limit its applicability to these age groups.

## Conclusions

This is the first study to validate the DHEQ-15-Sp for use in the Spanish population. The instrument has proven to be valid, reliable, and feasible for assessing dentine hypersensitivity. The results provide initial evidence that the DHEQ-15-Sp effectively measures the impact of dentine hypersensitivity on quality of life within the Spanish population.

## Figures and Tables

**Table 1:** The Spanish version of the DHEQ-15.

	Pensando sobre ti mismo, respecto al Ãºltimo mes hasta que punto estarÃ­as de acuerdo o en desacuerdo con las siguientes afirmaciones: (Thinking about yourself over the past month, to what extent would you agree or disagree with the following statements)
	(Por favor, marque solo una respuesta para cada pregunta) (Please tick only one response for each question)
1	Tener sensibilidad en mis dientes me quita mucho el placer de comer y beber. (Having sensations in my teeth takes a lot of the pleasure out of eating and drinking.)
2	Me lleva mucho tiempo terminar algunas comidas y bebidas por la sensibilidad en mis dientes. (It takes a long time to finish some foods and drinks because of sensations in my teeth.)
3	Ha habido momentos que he tenido problemas comiendo helado por esta sensibilidad. (There have been times when I have had problems eating ice cream because of these sensations.)
4	Tengo que cambiar la forma de comer o beber algunas cosas. (I have to change the way I eat or drink certain things.)
5	He de tener cuidado de cÃ³mo respiro en un dÃ­a frÃ­o. (I have to be careful how I breathe on a cold day.)
6	Cuando he comido algunas comidas me he asegurado de que no toquen algunos dientes. (When eating some foods I have made sure they don't touch certain teeth.)
7	Por esta sensibilidad, soy mÃ¡s lento comiendo que las otras personas que me acompaÃ±an. (Because of the sensations I take longer than others to finish a meal.)
8	He de tener cuidado de quÃ© como o bebo cuando estoy con otras personas a causa de la sensibilidad en mis dientes. (I have to be careful what I eat when I am with others because of the sensations in my teeth.)
9	Ir al dentista es molesto para mÃ­ porque sÃ© que va a ser doloroso a causa de la sensibilidad en mis dientes. (Going to the dentist is hard for me because I know it is going to be painful as a result of sensations in my teeth.)
10	Tengo ansiedad porque quizÃ¡ algo que yo coma o beba produzca sensibilidad en mis dientes. (I have been anxious that something I eat or drink might cause sensations in my teeth.)
11	La sensibilidad en mis dientes me enfada (como estado de Ã¡nimo). (The sensations in my teeth have been irritating.)
12	La sensibilidad en mis dientes ha estado molestÃ¡ndome (a nivel local de boca). (The sensations in my teeth have been annoying.)
13	Tener esta sensibilidad en mis dientes me hace sentir mayor. (Having these sensations in my teeth makes me feel old.)
14	Tener esta sensibilidad en mis dientes me hace sentir que los tengo lesionados. (Having these sensations in my teeth makes me feel damaged.)
15	Tener esta sensibilidad en mis dientes me hace sentir que mi salud general estÃ¡ afectada. (Having these sensations in my teeth makes me feel though I am unhealthy.)

**Table 2:** Sociodemographic Characteristics.

Variables		n	%
Gender	Female	114	55.6%
	Male	91	44.4%
Education level	No education	0	0%
	Primary education	9	4.4%
	Secondary education	7	3.4%
	High school	24	11.7%
	Vocational training	30	14.6%
	Higher education	135	65.8%

**Table 3:** Mean scores, corrected item-total correlations and Cronbach’s alpha for the Spanish version of DHEQ-15.

Item	Mean	Corrected item-total correlation	Cronbach´s Alpha if item is deleted
1	56.5	0.705	0.956
2	57.5	0.804	0.954
3	56.2	0.676	0.957
4	57.1	0.818	0.954
5	58.1	0.750	0.956
6	57.4	0.793	0.955
7	58.1	0.837	0.954
8	58.2	0.855	0,953
9	57.3	0.603	0.959
10	58.7	0.765	0.955
11	58.3	0.797	0.955
12	57.7	0.829	0.954
13	58.6	0.696	0.957
14	57.4	0.778	0.955
15	58.3	0.695	0.957

**Table 4:** Confirmatory Factor Analysis for 3 domains.

	Item	Restrictions-Coping	Identity	Social-Emotions
Restrictions	1	0.796	0.272	0.084
	2	0.780	0.301	0.278
	3	0.733	0.198	0.191
Coping	4	0.832	0.260	0.264
	5	0.626	0.282	0.415
	6	0.736	0.277	0.348
Social	7	0.678	0.371	0.411
	8	0.612	0.372	0.532
	9	0.225	0.152	0.853
Emotions	10	0.305	0.554	0.607
	11	0.384	0.526	0.584
	12	0.572	0.390	0.560
Identity	13	0.255	0.812	0.262
	14	0.489	0.705	0.197
	15	0.274	0.857	0.187

## Data Availability

The datasets used and/or analyzed during the current study are available from the corresponding author.
